# Early Diagnosis of Chemotherapy-Linked Cardiotoxicity in Breast Cancer Patients Using Conventional Biomarker Panel: A Prospective Study Protocol †

**DOI:** 10.3390/diagnostics12112714

**Published:** 2022-11-06

**Authors:** Saule Balmagambetova, Zhenisgul Tlegenova, Bekbolat Zholdin, Gulnara Kurmanalina, Iliada Talipova, Arip Koyshybaev, Dinara Nurmanova, Gulmira Sultanbekova, Mira Baspayeva, Saule Madinova, Kulparshan Kubenova, Ainel Urazova

**Affiliations:** 1 Department of Oncology, West Kazakhstan Marat Ospanov Medical University, 68 Maresyev Street, Aktobe 030019, Kazakhstan; 2 Department of Internal Diseases No. 2, West Kazakhstan Marat Ospanov Medical University, 68 Maresyev Street, Aktobe 030019, Kazakhstan; 3 Cardiology Division at University Medical Center, Building 8G, Zhanakonys, Aktobe 030017, Kazakhstan; 4 Chemotherapy Division at University Medical Center, Building 8G, Zhanakonys, Aktobe 030017, Kazakhstan; 5 Clinical Laboratory at University Medical Center, Building 8G, Zhanakonys, Aktobe 030017, Kazakhstan

**Keywords:** cardiotoxicity, chemotherapy, breast cancer, speckle tracking, biomarkers, kazakhstan

## Abstract

The prognosis of cancer treatment depends on, among other aspects, the cardiotoxicity of chemotherapy. This research aims to create a feasible algorithm for the early diagnosis of antitumor therapy cardiotoxicity in breast cancer patients. The paper represents a protocol for a prospective cohort study with N 120 eligible participants admitted for treatment with anthracyclines and/or trastuzumab. These patients will be allocated into four risk groups regarding potential cardiotoxic complications. Patients will be examined five times every three months for six biomarkers: cardiac troponin I (cTnI), brain natriuretic peptide (BNP), C-reactive protein (CRP), myeloperoxidase (MPO), galectin-3 (Gal-3), and D-dimer, simultaneously with echocardiographic methods, including speckle tracking. The adjusted relative risk (aOR) of interrupting an entire course of chemotherapy due to cardiotoxic events will be assessed using multiple analyses of proportional Cox risks. The Cox model will also assess associations between baseline biomarker values and time to cardiotoxic events. Moreover, partly conditional survival models will be applied to determine associations between repeated assessments of changes in biomarkers from baseline and time to cancer therapy-related cardiac dysfunction. All models will be adjusted for cancer therapy regimen, baseline LVEF, groups at risk, baseline biomarker values, and age. The decision-tree and principal component analysis (PCA) methods will also be applied. Thus, feasible patterns will be detected.

## 1. Introduction

Breast cancer (BC) was the most commonly diagnosed cancer in 2020 and remains as such as of 2021, accounting for 12% of all new annual cancer cases worldwide [1,2]. In Kazakhstan, the leading country in Central Asia, BC ranks second following lung cancer and accounts for 12.4% of all new cases as of 2021 [3]. Nevertheless, owing to cancer treatment achievements, the overall 5-year relative survival rate for breast cancer has reached 90% [4]. The prognosis of BC treatment depends on the tumor tissue’s histochemical properties, the tumor’s aggressiveness, the cancer process staging, and the cardiotoxicity of chemotherapy, now defined in the literature as cancer therapy-related cardiac dysfunction (CTRCD) [5]. At present, the CTRCD definition includes all possible cardiovascular complications occurring during anticancer treatment, such as left ventricular dysfunction, heart failure, myocardial infarction, arrhythmias or conduction disorders, acute myocarditis or pericarditis, hypertension arterial or hypotonia, and, in case of radiotherapy, coronary heart disease [6]. Currently, researchers distinguish two main typesof CTRCD: the anthracycline-associated, irreversible type with a cumulative effect, and the trastuzumab-induced, reversible, dose-independent type. Moreover, clinicians deal with its early-onset and late-onset varieties, as well as acute, subacute, and chronic forms [7,8]. As CTRCD can affect cancer survivors for years, decreasing their quality of life, the scope of the problem increases yearly. Only in the USA, 9.7 million females out of more than 18 million Americans with a history of cancer were alive on 1 January 2022, and the most prevalent tumor type was BC (4,055,770) [9]. A retrospective analysis of outcomes on all sarcoma patients in Denmark revealed that cardiotoxicity was observed in 31% of these patients. Of them, 70% experienced early-onset CTRCD [10].

The challenge of heart consequences after chemotherapy appeared so large-scale that it gave rise to a new subspecialty at the intersection of oncology and cardiology: cardio-oncology [11]. Cardio-oncologists primarily focused on echocardiographic methods in searching for methodsto detect CTRCD as early as possible. Transthoracic echocardiography (TTE) was recognized as the standard method to evaluate cardiotoxicity [12]. In the 2000s, the novel speckle tracking echocardiography (STE) method was widely introduced. STE allows for tracking of the displacement of “speckles” in two-dimensional (2D) echocardiographic images in an angle-independent way and assessment of their movement (strain) during the cardiac cycle. Its high feasibility, reproducibility, and accuracy have been demonstrated [13,14,15]. CTRCD is defined by either a decrease in left ventricular ejection fraction (LVEF) above 10% from baseline to a value of LVEF under 53%, or a decrease in global longitudinal strain (GLS) deformation below 15% from the baseline value [5,12,16]. Current echocardiography recommendations set the low normal value of 2D LVEF as 54% for women and 52% for men; hence, in the previous European Association of Cardiovascular Imaging (EACVI) position statement, a reduction in LVEF below 53% was classified as abnormal [17]. To date, researchers mainly focus on applying cardiac markers as they aresimple and relatively cost-effective compared to echocardiographic methods, particularly GLS assessment. Moreover, including biomarkers in the algorithms of CTRCD detection allows a comprehensive evaluation of cardiac function. Among biomarkers, cardiac troponins and B-type natriuretic peptide (BNP) seem to display the most proven efficacy [18,19].

Common practices include serial echocardiography and mostly troponin measurements. Nevertheless, although the pathophysiology of CTRCD has been characterized, there is currently no evidence-based approach for monitoring and managing patients who develop CTRCD [20]. Primarily, the task is to detect CTRCD at a preclinical stage, before any echocardiographic evidence of dysfunction or clinical symptoms are present, which would minimize cardiac complications. If the GLS assessment technique and three-dimensional (3D) echocardiography can cope with this task, the more affordable TTE cannot.

Therefore, searching for reliable biomarkers remains the research target regarding CTRCD’s feasible monitoring. Regrettably, to date, the cardio-oncologic integrative approach and development of appropriate tools to diagnose CTRCD conditions have not been implemented in the practice of Kazakhstani cardiologists and oncologists. Cardiac dysfunctions provoked by cancer treatment remain largely unexplored. The attitude to and awareness of CTRCD within involved specialties in Kazakhstan are similar to the state in many other countries. According to J. Peng et al. [21], the majority of cardiologists felt that cardiotoxicity should be monitored, even in asymptomatic cancer patients (55.8% vs. 12% of oncologists); the majority of cardiologists stated that cardio-oncology clinics would significantly improve cancer patients’ prognosis (88.3% vs. 45.8% of oncologists).

Thus, the current study is a first step toward forming a new subspecialty, cardio-oncology, in the country, by developing a program for the early diagnosis and timely correction of cardiotoxic complications of breast cancer antitumor therapy.

The rationale for this study is to use currently available methods to elaborate an algorithm to reveal chemotherapy-linked cardiotoxic complications early, to minimize their negative consequences. It is known that assessing the global longitudinal myocardial strain is the most effective technique in evaluating heart activity, although it isquite expensive. On the contrary, cardiac markers are considered the most cost-effective tools. Determining the predictive value of myocardial damage, inflammation, and oxidative stress biomarkers by assessing GLS in chemotherapy patients would allow the further application of these tests without combination with GLS, which is not always accessible. Detecting the incipient stages of cardiotoxicity using relatively cheap and the earliest-responding biomarkers would enable the development of a cost-effective algorithm for CTRCD’s timely correction. Generally, this research aims to identify steady patterns between the decrease in systolic myocardial function recorded by EchoCG, including speckle tracking, and the values of selected biomarkers.

In this context, we designed a multimarker panel focused chiefly on the tests’ affordability and relative comprehensiveness. The biomarkers’ values will be matched with EchoCG data (GLS) in definite periods. As such, we present an opportunity for the synchronous tracing of the changes and an evaluation of the comparative efficiency of all selected tests. The article presents a protocol for a prospective cohort study focused on biomarker research.

## 2. Materials and Methods

### 2.1. Protocol for Prospective Cohort Study

#### 2.1.1. Study Design

**Figure 1:**
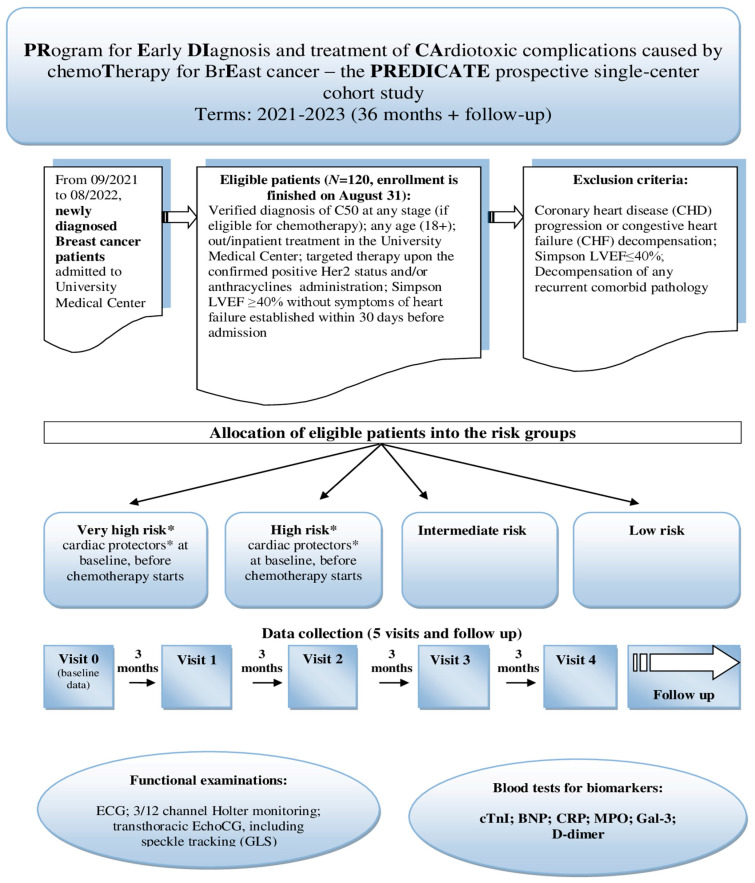
Design of the prospective cohort study.*“Very high” risk means presence of existing chronic heart failure or dilated cardiomyopathy, according to the current position papers on CTRCD [17,23,24,25]; “high” risk includes previous severe valvular heart disease, past myocardial infarction and/or revascularization, baseline left ventricular ejection fraction <50%, stable angina, or prior treatment with anthracyclines or radiation therapy (see Section 2.1.7). * Cardiac protectors: ACE inhibitors/ARBs; beta-blockers; statins; trimetazidine/analogues; others (see Section 2.1.5).

In this observational single-center study, a mixed design was used, with retrospective and prospective stages. The main points of the prospective cohort study are presented in Figure 1 (stage II). During the first, retrospective stage (from April to September 2021), we extracted all available information from the Aktobe Regional Oncologic Registry on BC patients who received chemotherapy with anthracyclines and/or targeted therapy in 2018–2019. The findings of this database research were published in 2022 [22]. Subsequently, survival rates from both samples (retrospective and prospective) will be matched within the framework of a quasi-experimental design.

#### 2.1.2. Study Population

At this stage, we consecutively recruited a single-center cohort of eligible women newly diagnosed with BC on admission to the chemotherapy division of the University’s Medical Center for treatment with anthracyclines an d/or trastuzumab. Given the single-center design, we chose the BC diagnosis due to its relatively high incidence, facilitating the recruitment of participants. The recruitment started on September 2021 and lasted until late August 2022. Criteria for participants’ eligibility are shown in Figure 1. Notably, the inclusion criteria do not imply participants who are administered targeted therapy with Kinase Inhibitors (KIs) or Immune Checkpoint Inhibitors (ICIs), only human epidermal growth factor receptor 2 (HER2)-positive individuals with indications of trastuzumab treatment. We also include patients who received radiation treatment before chemotherapy, as many schemes imply combined treatment. These patients will be allocated to a separate subgroup during analysis. The form of chemotherapy is also of significance. We select patients of any age (18+) and at any stage of BC but eligible for adjuvant or neoadjuvant chemotherapy, not palliative. Exclusion criteria are commonly accepted: patients showing LVEF ≤ 40% by Simpson or having established cardiac dysfunction or severe comorbidities are believed to be not eligible.

#### 2.1.3. Sample Size Estimation

To calculate the sample size for the prospective cohort, we analyzed data from the regional oncologic registry for ten years, 2010–2019, and literature sources on the incidence of LV dysfunction associated with chemotherapy [5,7,20]. The sample size was calculated using the Epi Info TM v.7 software (CDC USA) (https://www.cdc.gov/epiinfo (accessed on 2 September 2020)). We entered the following information: population size—150 (the average annual number of BC patients admitted to chemotherapy treatment at the University Medical center), the expected incidence of cardiovascular disease in women in the menopause transition aged 50+—9.7% [26], and the assessment accuracy—5%. The calculated sample size appeared to be equal to 100. Given the possible losses, the sample might be increased to 120 patients. As is known, the predictive model requires at least ten observations per predictor. We believe that the calculated sample size will be sufficient for a clinically relevant predictive model, as the resulting sample size allows for the detection of small-to-medium-sized group differences.

#### 2.1.4. Patient and Public Involvement

There is no patient or public involvement. There is no potential risk for patients due to the observational nature of this research.

#### 2.1.5. Data Collection and Timing

The research implies five participants’ visits, including an initial visit and one every three months (see Figure 1) Thus, the recruitment lasts twelve months, and the overall data collection will last until late August 2023. Given the follow-up, the prospective stage duration is thirty months. Data to be collected during visits are displayed in Table 1.

These data are listed in the patient’s individual registration card (IRC, Appendix No. 5 to the Protocol) and are entered into the dataset. The patient’s IRC example and other supplemental materials of the study are placed in a publicly available repository, osf.io [https://osf.io/nykmw/ (accessed on 10 June 2022)]. According to the Individual Participant Data Sharing Statement, Excel datasets would also be placed in this repository upon their completion (https://www.isrctn.com/ISRCTN12628444 (accessed on 21 July 2022).

#### 2.1.6. Definitions and Outcomes of the Study

The definition applied in the present study is as follows: CTRCD is defined by either a decrease in left ventricular ejection fraction (LVEF) above 10% from baseline to a value of LVEF under 53%, or a decrease in global longitudinal strain (GLS) deformation below 15% from the baseline value. Early cardiotoxicity is defined as <one year and late cardiotoxicity as ≥one year [5].

Accordingly, we apply the following definitions of measured outcomes.

- Primary outcomes:

1. The number of patients who develop cardiotoxic complications, including subclinical dysfunction during chemotherapy, measured using LVEF monitoring (<50% or >10% decline from baseline) and GLS assessment (decrease > 15% from baseline) at 12 months after the chemotherapy course started, through all groups at risk.

2. One-year survival without cardiotoxic complications, measured using LVEF monitoring and GLS assessment (as defined above) at 12 months after chemotherapy completion through all groups at risk.

In the primary outcomes, we included not only manifested CTRCD but also subclinical cardiac dysfunction during chemotherapy.

- Secondary outcomes:

Measured using biomarker measurement units on immunofluorescence analyzers at 3, 6, 9, and 12 months for all below:

1. Presence of increased values of the tests during chemotherapy treatment: cardiac troponin I (cTnI)—≥0.3 ng/mL; brain natriuretic peptide (BNP)—>100 pg/mL; C-reactive protein (CRP)—>5 mg/l; antibodies to myeloperoxidase (MPO)—>5 U/mL; galectin-3 (Gal-3)—>28.7 ng/mL; D-dimer—≥0.5 mg/L (presented values are taken from the analyzers’ manuals).

2. Time trends in the onset of increasing biomarker values in the LVEF and GLS data.

3. The predictive value of a positive result (PPV) and the predictive value of a negative result (PVN) for all enlisted biomarkers.

4. The number of all identified cardiovascular events during observation.

#### 2.1.7. The Risk Stratification Strategy

During baseline clinical examination (visit 0), the potential risk of CTRCD emergence is calculated. We use the strategy proposed in recent studies and adopted by current position papers on CTRCD [17,23,24,25], except for additional measurements after three–four medication cycles. According to the current strategy, the risk scores are calculated considering all possible risk factors—existing (recorded) cardiovascular diseases, the toxicity of chemotherapy prescribed, and lifestyle risk factors. If there is one intermediate risk factor or an absence of risks at the moment of examination, the patient is allocated to the low-risk group. If two to four intermediate risk factors are present, they are allocated to the intermediate risk group. If more than five intermediate risk factors or at least one high-risk factor are revealed, they are allocated to the high-risk group. Respectively, patients with one very high-risk factor are allocated to the very-high-risk group. Thus, all patients are divided into four risk groups, and patients from the groups of very high and high risk are provided with cardiac protectors from the pharmaceutical classes pointed out in Table 1. For all enrolled participants, a particular individual form, “Cardiovascular risk stratification for upcoming chemotherapy treatment,” is filled out (Appendix No. 6, [https://osf.io/nykmw/ (accessed on 10 June 2022)].) The patient’s risk scores will be calculated and stated in this form based on the listed possible risk factors and their defined hazard level and individual risk level.

#### 2.1.8. Outcome Measurements

General blood and biochemical tests for this study are performed according to the “Breast Cancer” in-country protocol dated 1 March 2019, No. 56, at the University Medical Center’s clinical lab. Some biomarkers’ detection (cTnI, BNP, CRP, D-dimer) is also performed in this lab using immunoassay analyzers and Finecare rapid quantitative tests, in line with Good Laboratory Practice (GLP). Galectin-3 and MPO assays were directed to a third-party collaborator, the “Olymp” labs network. The types of tests are the ELISA Galectin-3 S and ELiA MPO Well tests.

- Functional outcomes:

ECG machines and 24-record 12-channel wearable devices for outpatient Holter monitoring are used to detect rhythm and conduction disturbances, including the QT interval duration, in each enrolled participant during scheduled visits (*HM every six months, not three.) If cardiac complaints have emerged, out-of-schedule invitations are provided. Two project staffers are responsible for TTE and speckle tracking. Cardiac imaging is performed using “automated function imaging (AFI)—automatic non-Doppler assessment of longitudinal strain of the left ventricular myocardium” software and M5-SD transducer, 1.5–4.5 MHz. Left ventricular end-diastolic and end-systolic volumes are calculated using Simpson’s approach to derive LVEF. The same specialists also analyze GLS according to currently accepted protocols [17,27]. The study area is corrected to cover the whole thickness of the myocardial wall. Measurements are carried out from the apical 3-chamber (3C), 4-chamber (4C), and 2-chamber positions. When deriving apical positions, care is taken to ensure that the long axis of the ventricle is perpendicular to the plane of the mitral annulus in the LV apical views. Intra- and inter-observer coefficients of variation are not set for LVEF and GLS. CTRCD is defined as a ≥10% absolute decline in LVEF to a value of <53%. Subclinical LV myocardial dysfunction is considered to be a drop in GLS below (-) 18%, those in the range (0% to 17.9%), or a decrease in this indicator >15% from baseline. Of note, although we scheduled functional and laboratory measurements in terms of 0, 3, 6, 9, and 12 months, if patients receive the treatment on these dates, we postpone the tests until the chemotherapy cycle is completed or until the treatment interval. Usually, it takes up to 1–2 weeks.

#### 2.1.9. Adherence Control

It is known that poor adherence has been characterized as the most common cause of an unsatisfactory response to medication [28]. As the overwhelming majority of participants are not present in the inpatient division, we cannot monitor the number of pills (cardiac protectors) prescribed to patients from the two high-risk groups or use other vigorous methods of compliance control. Instead, we practice monthly encouraging calls to patients under observation to ensure retention and adherence to protocol activities. Moreover, during the baseline examination, we assist willing participants who are prescribed cardiac protectors in installing appropriate apps on their telephones to remind them to take their pills. There are two project staffers responsible for these activities. According to research on experience in the discussed issue, 89% adherence (medication taken) under long-term observation is recognized as an excellent result [29].

### 2.2. Statistical Analysis

In this study, baseline characteristics will be summarized using proportions for categorical variables (cancer treatment, revealed cardiotoxic complications, etc.), and medians (interquartile range) be presented for continuous variables (biomarker values, etc.). The Pearson χ^2^ criterion will be applied to identify intergroup differences for categorical variables. Quantitative variables will be compared using the nonparametric U Mann–Whitney test in terms of rhythm disturbances (ECG/HM), dynamics in the level of biomarkers (0, 3, 6, 9, 12 months), the degree of myocardial dysfunction identified by LVEF and GLS (0, 3, 6, 9, 12 months), etc., for two unrelated groups (with and without the development of cardiotoxicity.) A comparison of three or more independent groups (e.g., four risk groups) will be carried out using parametric analysis of variance (one-way ANOVA) or a nonparametric Kruskal–Wallis H-test. For repeated measurements (related samples), analysis of variance with Greenhouse–Geisser corrections will be applied.

To characterize the changes in biomarker levels according to treatment groups, mean estimated changes from baseline will be plotted over time. Considering radiation treatment received immediately before the chemotherapy as a source of potential bias, we arrange not only anthracycline, trastuzumab, and combined treatment groups but also a separate subgroup after radiation treatment. Mean changes will be determined using repeated-measures linear regression estimated via generalized equations. Each model will be adjusted for the baseline values of the biomarker under consideration and the time since the treatment started. Contemporaneous associations between changes in biomarkers from baseline and changes in LVEF will also be determined using repeated-measures linear regression.

To assess the influence of independent factors on the binary variable of response (cardiotoxicity), multiple logistic regression analysis (LRA) will be used by the sequential exclusion of variables (age, biomarkers, arrhythmias, LVEF according to Simpson and GLS data, etc.). The presence of a statistically significant relationship with the predicted event in the one-dimensional analysis will be the criterion for inclusion in the multivariate analysis. The results will be presented as unadjusted (uOR), adjusted odds ratios (aOR), and 95% CI. The incidence of cardiotoxic complications of chemotherapy will also be presented with the 95% CI, calculated according to the Wilson method. Sensitivity analysis (the Cornfield method) will also be applied for the binary variable of response (cardiotoxicity).

Indices of sensitivity (Sn), specificity (Sp), PPV, and PVN will be calculated for each diagnostic test under consideration (biomarkers, TTE, rhythm disturbances, etc.), as well as for predictive models in general.

The risk of interrupting a course of chemotherapy due to cardiotoxicity in the analyzed groups will be assessed through Kaplan–Meier survival curves. Given the influence of potential confounders, the relative risk of interrupting an entire course of chemotherapy due to cardiotoxic events will be assessed using multiple analyses of proportional Cox risks. The results will be presented as aOR with a 95% CI. Associations between baseline biomarker values and time to CTRCD will also be assessed using Cox proportional hazards model. Moreover, partly conditional survival models will be applied to determine associations between repeated assessments of changes in biomarkers from baseline and time to CTRCD. All models will be adjusted for cancer therapy regimen, baseline LVEF and group at risk (and cardiac protection if administered), baseline biomarker values, age, comorbidities index, and body mass index. Associations between changes from baseline in biomarker levels over time and subsequent changes in LVEF and longitudinal strain will be assessed similarly. Differences in the associations between changes in biomarkers and LVEF/GLS across the different treatment groups will be evaluated by including biomarker–treatment interaction terms, as shown in the recent paper by Demissei et al. [30].

Due to the number of variables subjected to analysis, and the anticipated presence of multiple outcomes, the included tests may not be sufficient to solve the task of building a clinically relevant decision algorithm, and so the decision-tree method will be applied. Decision trees break down complex data into more manageable parts, which is beneficial in prediction analysis, data classification, and regression. A dimensionalityreduction method, principal component analysis (PCA), will also be applied.

Two-sided levels <0.05 are assumed to be statistically significant. For statistical processing, software packages SPSS (IBM, Armonk, NY, USA, v.25), Statistica (StatSoft, Inc., Tulsa, OK, USA, v. 10), and R 3.3.2 (R Foundation for Statistical Computing, Vienna, Austria).

### 2.3. Study Ethics and Dissemination Plan

As mentioned above, this study poses no risk to the participating individuals. Study participation does not imply restrictions in receiving any clinical care determined by oncologists (additional CT, MRI, etc.). All study procedures are conducted according to the principles of the Declaration of Helsinki (2013), and patient rights are observed. Participation in the study is voluntary, and participants can leave the study at any time for any reason. This provision was stated in the informed consent form at enrollment in the study *(*https://osf.io/nykmw/(accessed on 10 June 2022). Subsequent publications will include the interim research results and final results with developed algorithms (in peer-reviewed journals). Moreover, the dissemination plan permits the preparation of country-scale clinical recommendations on the management of CTRCD for cardiologists and oncologists.

## 3. Preliminary Results of the Study

Previous retrospective research for 2018–2019, the first stage of the current project, aimed to clarify the proportion and structure of complications that led to chemotherapy interruption in BC patients. This registry study included all available characteristics of the oncologic process: tumor staging and histological type, medications used, duration of chemotherapy, occurring complications, treatment outcomes, and survival rates.

Overall, 305 BC cases were analyzed. Chemotherapy was completed successfully in 65.9% of patients and interrupted in 10.5% due to adverse events. Cardiovascular disorders were identified in 6.2%. There were significant differences in the number of detected cardiovascular complications between the two groups of patients—those under EchoCG monitoring and those without (*p* < 0.001)—but no difference in survival was found in the two groups (*p* 0.814). Survival in patients with cardiovascular complications was 28.1 months, compared to 34.3 months in the group without CV events (*p* < 0.005). Those who completed a full course of chemotherapy had a survival rate of 34.9 months, compared to 17.6 months in persons whose treatment was interrupted due to complications (*p* < 0.001). We identified four fatal cases of cardiotoxicity that were not recorded in the oncologic registry as CTRCD.

## 4. Discussion

In the mentioned preface to the cohort study (retrospective research for 2018–2019), we obtained an idea of CTRCD’s scope in the region, establishing a 6.2% CTRCD prevalence and reporting four fatal cases [22]. Analysis of these cases revealed that only one of the women was administered cardiac protectors, and three women received adjuvant radiation treatment before admission to chemotherapy. Breast cancer patients who have undergone even small-dose radiation, particularly left-sided exposure, are known to carry an increased risk of ischemic heart disease, which is proportional to the radiation dose [31,32]. In the present cohort study, we cannot exclude those who underwent radiation treatment before chemotherapy due to the relatively small number of eligible patients in a single-center setting. This circumstance can lead to bias, and we will manage these patients as a separate subgroup during analysis, as stated above. Recent research reported the cardiotoxicity risk of relatively new classes of targeted therapies such as KIs or ICIs [33,34,35,36]. We exclude those who are administered the mentioned treatments to minimize the subgroups under analysis.

A great deal of research has proposed and tested different panels, combining various markers. Emerging biomarkers include myeloperoxidase (MPO), placental growth factor (PIGF), growth differentiation factor 15 (GDF-15), C-reactive protein (CRP), Galectin-3 (Gal-3), microRNAs, etc., having potential value in predicting CTRCD before any signs of overt cardiotoxicity are apparent [37,38]. Some of these biomarkers (CRP, Gal-3, MPO) reflect such aspects of heart pathophysiology as oxidative stress, inflammation, and fibrosis [39]. A high serum level of CRP (>10 mg/L) is known to predict mortality in patients with acute decompensated heart failure (HF) one year after discharge [40]. CRP has recently been shown to have important predictive value in tumor immunotherapy. CRP is a prognostic biomarker for Immune Checkpoint Inhibitor (ICI) treatment [41,42]. Gal-3, a member of the beta-galactoside-binding lectin family, is involved in the occurrence and development of cardiac fibrosis, HF, and atherosclerosis [43]. In addition, Gal-3 inhibitors can effectively block lung adenocarcinoma growth and metastasis and increase the efficacy of PD-L1 ICIs [44]. Moreover, Gal-3 concentrations appear to increase before heart failure manifests, making it a potential screening tool for patients at risk of heart failure [45]. MPO is known to be an independent predictive factor of 1-year mortality in acute HF patients [46]. D-dimer is a sensitive biomarker for cancer-associated thrombosis, but little is known about its significance in CTRCD. Japanese researchers recently established that the occurrence of CTRCD was higher in the high D-dimer group compared to the low group (16.2 vs. 4.5%, *p* = 0.0146, *n* 169) [47].

Undoubtedly, high-sensitivity cardiac troponin T (hs-cTnT) and NT-proBNP (N-terminal pro-B-type natriuretic peptide) are currently recognized as the most effective predictors of CTRCD [18,19,30,48]. According to a recent review, only NPs (BNP and NT-proBNP) come close to the characteristics of “ideal” HF biomarkers. They are often regarded as the reference standard against which other potential biomarkers must be evaluated, as they may allow the identification of patients with subclinical LV dysfunction [49]. Compared with BNP, NT-proBNP has a much broader “gray zone”. Notwithstanding, these hormones are clinically equivalent in diagnosing CHF, and recalculation is possible [50,51,52]. Presumably, BNP appears more convenient in many actual situations. Moreover, a definite correlation between D-dimer and BNP is found in patients with various pathologies. However, this can lead to an incorrect diagnosis regarding suspected cases of hypercoagulable states in heart failure. More clinical data are needed to rely on such a correlation [53,54].

Troponin test elevation occurs even in chronic heart failure patients as an indicator of myocardial stress. Within the panel of troponins, hsTn assays are believed to be the most sensitive tests. Nevertheless, there is a loss of specificity unless specific protocols, particularly those involving “deltas”, are used [55,56]. Moreover, researchers found that when using hsTnI, a sex-specific threshold for MI diagnosis doubled the diagnosis of MI in female participants [57]. Meanwhile, recently, the longitudinal LVEF trajectory, but not hs-cTnT or NT-proBNP, was reported as allowing for a dynamic assessment of cardiotoxicity risk in early BC [58]. Thus, an evidence-based approach for managing CTRCD patients is yet to be developed.

### Strengths and Limitations of This Study

- This study is the first of its kind across Kazakhstan and Central Asia, thus creating a springboard for future developments in cardio-oncology.
- All biomarkers selected for the panel are relatively affordable and can provide a comprehensive assessment of cardiovascular disorders at definite periods over two years of CTRCD monitoring in cancer patients, irrespective of cancer nosology.
- The single-center design may be considered the study’s main limitation, resulting in a relatively small sample size compared to other researchers’ data and potentially affecting the results’ generalizability.
- One of the limitations potentially leading to bias is the impossibility of excluding those who receive radiation treatment before chemotherapy.
- The absence of set intra- and inter-observer coefficients of variation for LVEF and GLS may also be referred to as a limitation of this study.

## 5. Conclusions

The PREDICATE research aims to study the effect of chemotherapy on myocardial function and structure, presenting groups of BC patients for whom GLS assessment and a proposed panel of biomarkers (cTnI, BNP, D-dimer, CRP, MPO, and Gal-3) would be the most appropriate for the timely diagnosis and treatment of cardiotoxicity. The selected tests were not combined in previous research by other authors. Some of these biomarkers are included in routine examinations and do not require additional financing. We believe that it would be helpful to establish further patterns in their relationship at certain time intervals. In the future, the involvement of other in-country clinics to enlarge the number of participants under CTRCD monitoring is needed.

## Figures and Tables

**Table 1:** Type of data subject to collection during visits.

Domain	Measures (Itemized)
General information	Patient’s code (individual identification No.); age; ethnicity; main occupation for the last 12 months; menopause; BMI*
Breast cancer features	Tumor clinical staging; tumor histotype; tumor clinical classification; IHC* data
Presence of risk factors	BC hereditary factor; smoking; physical activity (hour, min); history of stroke; IHD*, CHF* (functional class); Charlson comorbidity index (disease, scores); arterial hypertension; diabetes mellitus (presence/absence)
Clinical examination at admission	Heart rate; SBP*; DBP*; 6-min walk test (functional class, meters); selected clinical and biochemical lab tests; ECG* and Holter monitoring (HM every 6 months)
Breast cancer treatment	Medications prescribed (dose, regimen) Previous treatment, before admission to chemotherapy division: radiation (dose, regimen), surgical (radical, sectoral)
Cardiac protectors prescribed	ACE* inhibitors/ARBs*; beta-blockers; statins; trimetazidine/analogues; others
Echocardiographic data and biomarkers	See Figure 1 (including time periods of change onset)
Complications of Chemotherapy	
CTRCD conditions	Code; type; form; time of emergence
Other complications	Hematopoietic system complications; thromboembolic complications; pericarditis; gastrointestinal tract; respiratory system; urinary system; allergic reactions; neurotoxicity; toxic effects on the skin and appendages; toxic hyperthermic reactions; toxic phlebitis
Chemotherapy outcomes	Without complications; CT* correction due to CTRCD; CT interruption due to CTRCD; CT correction due to non-CTRCD complications; CT interruption due to non-CTRCD complications
Completeness of chemotherapy	(%)
Death	(Cause; date)
Dropped out of the study	Reason

*ACE—angiotensin-converting enzyme inhibitors; *ARBs—angiotensin II receptor blockers; *BMI—body mass index; *CHF—chronic heart failure; *CT—chemotherapy; *ECG—electrocardiogram; *IHC—immunohistochemical test; *IHD—ischemic heart disease; *SBP, *DBP—systolic, diastolic blood pressure.

## Data Availability

The data obtained in this study will be openly available at [https://www.isrctn.com/ISRCTN12628444 (accessed on 21 July 2022)] and [https://osf.io/nykmw/(accessed on 10 June 2022)].

## Supplementary Materials

As mentioned in the text, all supplementary materials relevant to the presented study protocol are placed in a publicly available repository: [https://osf.io/nykmw/ (accessed on 10 June 2022)]. The STROBE checklist for cohort studies is added as a supplementary file.
