# Supplementary material for: Early Diagnosis of Chemotherapy-Linked Cardiotoxicity in Breast Cancer Patients Using Conventional Biomarker Panel: A Prospective Study Protocol
Source: Diagnostics (Basel). 2022 Nov 6;12(11):2714. doi: 10.3390/diagnostics12112714 (PMC9689308; doi:10.3390/diagnostics12112714)
Supplement: Supplementary file 1 [file diagnostics-12-02714-s001.zip › diagnostics-1962418-supplementary.pdf]

**INDIVIDUAL REGISTRATION CARD (IRC)**

**Appendix No. 5**

STUDY "DEVELOPMENT OF A PROGRAM FOR EARLY DIAGNOSTICS AND TREATMENT OF CARDIOTOXIC COMPLICATIONS CAUSED BY CHEMOTHERAPY OF BREAST CANCER"

RESEARCHER ___________________ Recruitment date _________

**Patient's code** ____________________ Indiv. identification number __________________________

Phone ___________________________

Address ___________________________________________________________________________

**Inclusion Criteria:**
Age 18 years and older;
Outpatient or inpatient treatment at the University's Medical Center with a verified diagnosis of breast cancer of any stage;
Administration of targeted therapy in case of confirmed Her2 positive status and/or drugs from the Anthracycline group.

**Exclusion Criteria:**
A history of the cardiotoxic effect of chemotherapy, regardless of the statute of limitations;
Progression of coronary artery disease or decompensation of CHF in the previous 6 months;
Simpson left ventricular ejection fraction ≤40%;
Decompensation of comorbid pathology (endocrine disorders, diseases of the liver, kidneys, bronchopulmonary system) in the previous 3 months.

**GENERAL INFORMATION:**

**Name** ____________________________________________________________________

**Date of Birth:** ______________________ Age ______________ Gender ______________

***Ethnicity:*** Asian - 1; European - 2; I refuse to answer - 3.

***Marital status:*** Single/Not married – 1; Married – 2; Divorced -3; Widow/widower – 4;
I refuse to answer – 5.

***Occupation (main occupation for the last 12 months):***
State employee -1; Private sector employee -2; Entrepreneur - 3; Student - 4; Housewife - 5; Retired - 6; Unemployed (able to work) - 7; Disabled - 8; Refuse to answer - 9.

Height___________ (см); Weight _____________(kg); BMI______________(kg/м$^2$)

**I.BREAST CANCER FEATURES**

***Hereditary factor:*** Yes - 1; No - 2; ***Menopause:*** Yes - 1; No - 2; **BC localization:** Right - 1; Left – 2;

***Clinical staging:*** 1 – I St; 2 – IIA St; 3 – IIB St; 4 – IIIA St; 5 – IIIB St; 6 - IV St.

***Tumor histotype*:**
1 - invasive carcinoma without specification;
2 - intraductal cancer;
3 - infiltrating ductal cancer;
4 - lobular cancer;
5 - carcinosarcoma.

***Clinical classification of the tumor:***
1 - nodal cancer
2 - diffuse form - mastitis-like cancer
3 - diffuse form - edematous-infiltrative cancer
4 - diffuse form - Paget's cancer
5 - diffuse form - shell cancer
6 - diffuse form - erysipelatous cancer
7 - diffuse form - others, without specification.

***Immunohistochemical data:***
1 – Triple negative TNBC ( ER (-), PR (-), Her-2-neu – neg, Ki 67 – any);
2 – Luminal type A ( ER (+) and/or PR (+), Her-2-neu – neg., Ki 67 – < 20%);
3 – Luminal B type (positive) (ER (+) and/or PR (+), Her-2-neu – pos., Ki 67 – any);
4 – Luminal B type (negative) тип (ER (+) and/or PR (+), Her-2-neu – neg., Ki 67 – > 20%);
5 – Her-2-positive type (ER (-), PR (-), Her-2-neu – pos., Ki 67 – any).

**II.VISITS**

| **Factors** | **Visit 0** | **Visit 1** | **Visit 2** | **Visit 3** | **Visit 4** |
|---|---|---|---|---|---|
| Smoking, yes-1, no-2 | | | | | |
| Physical activity (hour, min) | | | | | |
| SBP | | | | | |
| DBP | | | | | |
| Heart rate, beats/min | | | | | |
| Hemoglobin, g/l | | | | | |
| Leukocytes, $10^9$ | | | | | |
| Platelets, $10^9$ | | | | | |
| Blood glucose, mmol/l | | | | | |
| Creatinine, mmol/l | | | | | |
| Bilirubin, mmol/l | | | | | |
| Total cholesterol, mol/l | | | | | |
| Arterial hypertension, yes-1, no - 2 | | | | | |
| Diabetes mellitus, yes-1, no - 2 | | | | | |
| IHD (functional class I,II,III,IV) | | | | | |
| CHF (functional class I,II,III,IV) | | | | | |
| History of stroke | | | | | |
| CHARLSON, scores | | | | | |
| 6-minute walk test, meters | | | | | |
| 6-minute walk test,<br>functional class I,II,III,IV | | | | | |
| **Cardiac protectors** | | | | | |
| 1ACE inhibitor/ARA blockers | | | | | |
| 2Beta-blockers | | | | | |

| | | | | | |
|---|---|---|---|---|---|
| 3Calcium channel blockers | | | | | |
| 4Statins | | | | | |
| 5Trimetazidine and analogues | | | | | |
| 6 Other (write in) | | | | | |
| 7Other (write in) | | | | | |
| **BC treatment** | | | | | |
| Doxorubicin, dose/regimen | | | | | |
| Trastuzumab, dose/regimen | | | | | |
| Radiation therapy, dose/regimen | | | | | |
| Surgical treatment:<br>Radical -1;<br>Sectoral-2 | | | | | |
| **Biomarkers** | | | | | |
| cTn, ng/mL | | | | | |
| BNP, pg/mL | | | | | |
| CRP, mg/L | | | | | |
| D dimer, mg/L | | | | | |
| MPO, U/ml | | | | | |
| Gal-3, ng/ml | | | | | |
| **ECG, HM, EchoCG** | | | | | |
| ECG, code | | | | | |
| Holter monitoring*, code | | | | | |
| LVEF (Simpson), % | | | | | |
| GLS, % | | | | | |
| Cardiotoxicity type, code | | | | | |
| * - 0,2,4 visits | | | | | |

**CHARLSON Comorbidity index**

| DISEASES | SCORES |
|---|---|
| Myocardial infarction | 1 |
| Chronic heart failure | 1 |
| Peripheral artery disease | 1 |
| Cerebrovascular disease | 1 |
| Dementia | 1 |
| Chronic lung disease | 1 |
| Connective tissue disease | 1 |
| Peptic ulcer | 1 |
| Mild liver damage | 1 |
| Diabetes | 1 |
| Hemiplegia | 2 |
| Moderate or severe kidney disease | 2 |
| Diabetes mellitus with organ damage | 2 |
| Malignant tumor without metastases | 2 |
| Leukemia | 2 |
| Lymphomas | 2 |
| Moderate or severe liver damage | 3 |
| Metastatic malignant tumors | 6 |
| AIDS (disease, not viremia) | 6 |

| | |
|---|---|
| Age | 40-49 years old - 1 score; 50-59 years old - 2 scores; 60-69 years old - 3 scores; 70-79 years old - 4 scores, etc. |
| **CHARLSON comorbidity index, scores** | |

**6-minute walk test**

| Parameter | initially | After test |
|---|---|---|
| Shortness of breath (Borg scale) | | |
| Weakness (Borg scale) | | |
| Heart rate | | |
| Arterial BP | | |

Pauses or stops during the test: yes / no; time__________amount _____________
Symptoms during the test: angina pectoris _____________ dizziness __________
leg pain __________ weakness _________ other __________________________
Number of laps_____________________________ Distance covered__________

**FC score NYHA**

| The severity of CHF (NYHA) | Distance 6-minute walk |
|---|---|
| I FC | 426-550 |
| II FC | 301-425 |
| III FC | 151-300 |
| IV FC | <150 |

**Complications of Chemotherapy**

| **Factors (code)** | **Visit 0** | **Visit 1** | **Visit 2** | **Visit 3** | **Visit 4** |
|---|---|---|---|---|---|
| 1 Cardiotoxic (code) | | | | | |
| 2 Hematopoietic system | | | | | |
| 3 Thromboembolic complications | | | | | |
| 4 Pericarditis | | | | | |
| 5 Gastrointestinal tract | | | | | |
| 6 Respiratory system | | | | | |
| 7 Urinary system | | | | | |
| 8 Allergic reactions | | | | | |
| 9 Neurotoxicity | | | | | |
| 10 Toxic effect on the skin and appendages | | | | | |
| 11 Toxic hyperthermic reactions | | | | | |
| 12 Toxic phlebitis | | | | | |

**Chemotherapy outcomes**

| **Factors (code)** | **Visit 0** | **Visit 1** | **Visit 2** | **Visit 3** | **Visit 4** |
|---|---|---|---|---|---|
| 1 According to the plan, without complications | | | | | |
| 2 ChT correction due to CT | | | | | |

| | | | | | |
|---|---|---|---|---|---|
| complications | | | | | |
| 3 ChT suspension due to CT complications | | | | | |
| 4 Correction of CT due to non-CT complications | | | | | |
| 5 ChT suspension due to non-CT complications | | | | | |
| 6 Completion of chemotherapy, % | | | | | |
| 7 Death (date, cause) | | | | | |
| 8 Dropped out of the study | | | | | |

**Codes for ECG and Holter monitoring**

**ECG codes**
1 - Norm;
2 - Sinus tachycardia (HR> 90 beats / min);
3 - Sinus bradycardia (HR<60 bpm);
4 - Atrial extrasystoles;
5 - Ventricular extrasystoles;
6 - Atrial fibrillation
7 - AVB I st, AVB II st Mobitz 1;
8 - AVB II st Mobitz 2, AVB III st;
9 - Bundle branch block;
10- ST segment depression/elevation;
11- Prolongation of QT corrected.

**HMECG codes**
1-Norm;
2-Atrial extrasystoles single, paired;
3-Atrial extrasystoles group, paroxysms of atrial tachycardia;
4-Ventricular extrasystoles Laun I, II, III;
5- Ventricular extrasystoles Laun IV, V;
6-Atrial fibrillation;
7-AVB Ist, AVB II st Mobitz 1;
8-AVB II st Mobitz 2, AVB IIIst;
9- Bundle branch block;
10-ST segment depression/elevation;
11- Corrected QT prolongation.

**Complications of Chemotherapy (codes)**

**Cardiotoxic (codes):**
1-Subclinical LV dysfunction;
2-Clinical LV dysfunction;
3-Rhythm and conduction disorders;
4-Arterial hypertension
5-Angina pectoris;
6-Pulmonary hypertension;
7-Pericarditis;
8-Thrombosis and thromboembolism;
9-Peripheral vascular disease and stroke

**Type of cardiotoxicity:**
A-Acute;
B-Subacute;
C-Chronic

**Hematopoietic system:**
1 - Anemia
2 - Thrombocytopenia
3 - Leukopenia
Note_____________________________________

**Gastrointestinal tract**
1- Nausea and vomiting
2 - Stomatitis/mucositis
3 - Toxic enterocolitis
4 - Toxic hepatitis

**Respiratory system**
1-Acute respiratory failure
2-Bronchospasm
3-Pneumonitis, bronchitis
4-Pulmonary edema
Note_________________________________________

**Urinary system**
1-Nephrotoxicity
2-Toxic cystitis
Note_________________________________________

**Allergic reactions**
1-Anaphylactic shock
2-Urticaria

3-Bronchospasm
Note_____________________________________

**Neurotoxicity**
1-Peripheral
2-Central
3- Psychovegetative disorders
Note_____________________________________

**CARDIOVASCULAR RISK STRATIFICATION**
**FOR UPCOMING CHEMOTHERAPY TREATMENT**

**Appendix No. 6**

Study "DEVELOPMENT OF A PROGRAM FOR EARLY DIAGNOSIS AND TREATMENT OF CARDIOTOXIC COMPLICATIONS CAUSED BY BREAST CANCER CHEMOTHERAPY" (PREDICATE)

PATIENTS' CODE____________________ DATE OF FILLING IN _____________________

| RISK FACTOR | HAZARD LEVEL OF THE RISK FACTOR | PATIENT'S RISK LEVEL |
|---|---|---|
| Existing chronic heart failure or dilated cardiomyopathy | Very high | |
| Previous severe valvular heart disease | High | |
| Past myocardial infarction and/or revascularization | High | |
| Stable angina | High | |
| Baseline left ventricular ejection fraction <50% | High | |
| Initial left ventricular ejection fraction 50-54% | Intermediate | |
| Elevated Troponin at baseline | Intermediate | |
| Initially elevated NT-proBNP or BNP | Intermediate | |
| Age over 80 | High | |
| Age 65-79 years | Intermediate | |
| Arterial hypertension | Intermediate | |
| Diabetes mellitus | Intermediate | |
| Chronic kidney disease | Intermediate | |
| Prior treatment with Anthracyclines | High | |
| Prior Radiation Therapy | High | |
| Prior anticancer therapy without Anthracyclines | Intermediate | |
| Long history of smoking | Intermediate | |
| Obesity with body mass index >30 $kg/m^2$ | Intermediate | |
| *TOTAL RISK OF CARDIOTOXIC COMPLICATIONS* | | |

*Note:
Low risk of cardiotoxicity (CT) - 1 intermediate risk factor (RF) or no RF;
Intermediate risk of CT - from 2 to 4 intermediate risk factors;
High risk CT - more than 5 intermediate risk factors or 1 high risk factor;
Very high risk CT - presence of very high risk factor.
